# A PIK3CA-mutant breast cancer metastatic patient-derived organoid approach to evaluate alpelisib treatment for multiple secondary lesions

**DOI:** 10.1186/s12943-022-01617-6

**Published:** 2022-07-22

**Authors:** Sara Donzelli, Mario Cioce, Andrea Sacconi, Francesca Zanconato, Theodora Daralioti, Frauke Goeman, Giulia Orlandi, Simona Di Martino, Vito Michele Fazio, Gabriele Alessandrini, Stefano Telera, Mariantonia Carosi, Gennaro Ciliberto, Claudio Botti, Sabrina Strano, Stefano Piccolo, Giovanni Blandino

**Affiliations:** 1 Translational Oncology Research Unit, IRCCS Regina Elena National Cancer Institute, Rome, Italy; 2 Laboratory of Molecular Medicine and Biotechnology, University Campus Bio-Medico of Rome, Rome, Italy; 3 Institute of Translational Pharmacology, National Research Council of Italy (CNR), Rome, Italy; 4 Clinical Trial Center, Biostatistics and Bioinformatics Unit, IRCCS Regina Elena National Cancer Institute, Rome, Italy; 5 Present Address: Department of Molecular Medicine, University of Padua, Padua, Italy; 6 Department of Pathology, IRCCS Regina Elena National Cancer Institute, Rome, Italy; 7 SAFU Unit, IRCCS Regina Elena National Cancer Institute, Rome, Italy; 8 Scientific Direction, IRCCS San Gallicano Dermatological Institute, Rome, Italy; 9 Department of Pathology, Biobank, IRCCS Regina Elena National Cancer Institute, Rome, Italy; 10 Laboratory of Oncology, Fondazione IRCCS Casa Sollievo della Sofferenza, San Giovanni Rotondo, 71013 Italy; 11 Thoracic Surgery, IRCCS Regina Elena National Cancer Institute, Rome, Italy; 12 Neurosurgery Unit, IRCCS Regina Elena National Cancer Institute, Rome, Italy; 13 Scientific Direction Office, IRCCS Regina Elena National Cancer Institute, Rome, Italy; 14 Breast Surgery Unit, IRCCS Regina Elena National Cancer Institute, Rome, Italy; 15 IFOM ETS, the AIRC Institute of Molecular Oncology, Milan, Italy

**Keywords:** Breast cancer metastases, Organoids, Alpelisib, *PIK3CA* mutations

## Introduction

Breast cancer (BC) is a powerful example of the intra- and interpatient heterogeneity of tumours; thus, there remain several grey areas in BC treatment approaches. This is especially true for advanced/metastatic disease (mBC) [1–3]. Organoid cultures are suitable models for studying the histological complexity and genetic heterogeneity of parental tumours and can be used to assess treatment strategies for challenging diseases such as mBC. Here, we report a proof-of-concept test case using organoid cultures from metastatic BC specimens to rapidly test drug sensitivity and molecular-lesion-driven treatments. Based on the relevant impact of *PIK3CA* gene mutations on breast cancer progression and resistance to therapy [4], we assessed the efficacy of alpelisib (BYL719; Novartis Pharma AG, Basel, Switzerland), a specific PI3K-α inhibitor, on organoid cultures derived from multisite mBC samples carrying specific *PIK3CA* gene mutations.

## Results

**Fig. 1:**
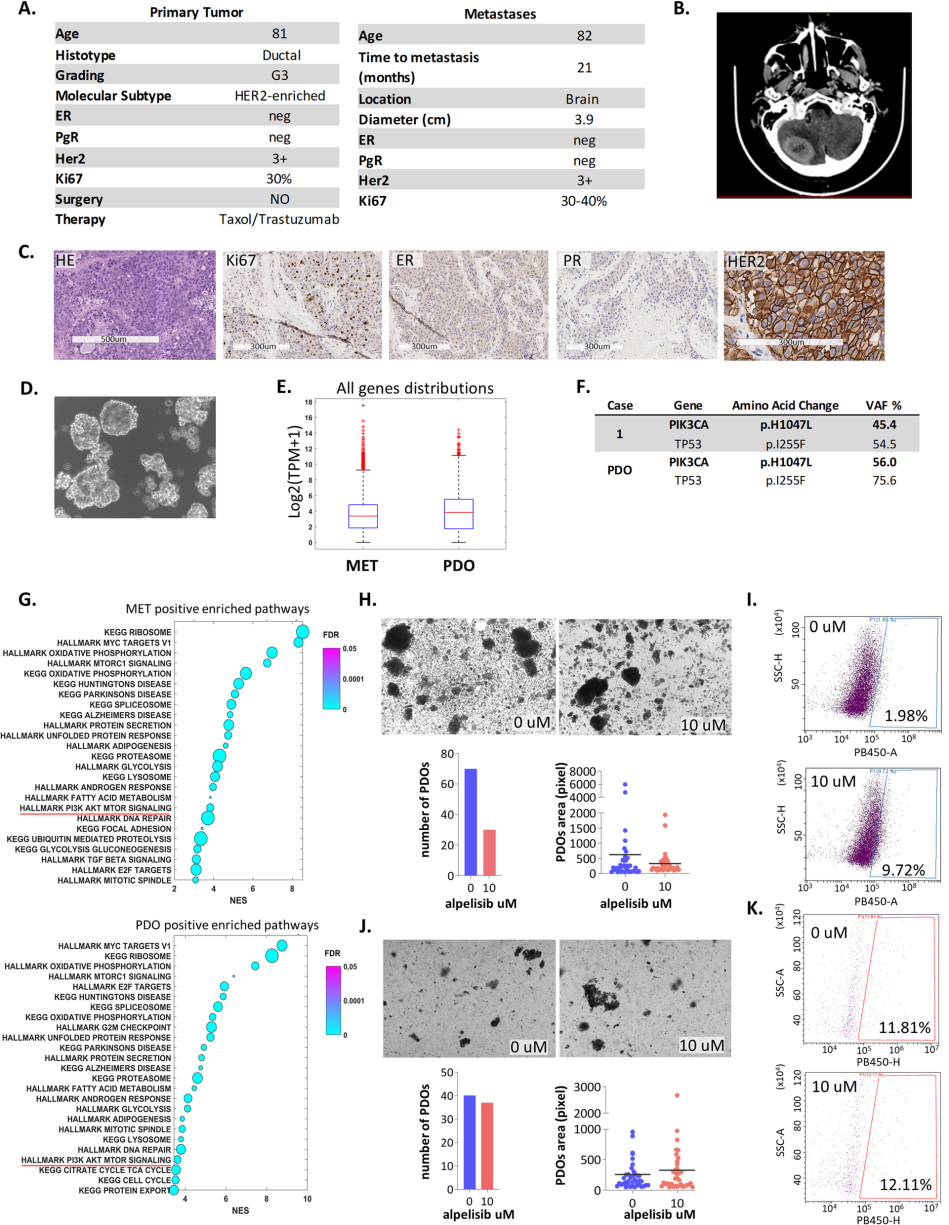
**A** Clinical features of case 1: primary breast cancer and relative brain metastases. **B** Contrast-enhanced brain computed tomography scan showing a solid and enhanced large lesion (3.9 cm) in the cerebellum with perilesional oedema. **C** Representative staining results from haematoxylin–eosin (HE) and immunohistochemistry for ER, PR, HER2 and Ki-67 in the metastatic lesion from patient 1. Scale bar = 200–500 µM. ER, oestrogen receptor; PR, progesterone receptor; HER2, human epidermal growth factor. **D** Representative bright-field microscopy image of organoids generated from metastatic lesions. Magnification: 20X. **E** Summary of the mutational profile of case 1 brain metastases and related organoids. VAF: variant allele frequency. **F** All expressed gene distributions by RNA-seq analysis of metastatic lesions (METs) and related organoids (PDOs). RNAseq data counts were normalized as transcripts per million (TPM). **G** Preranked gene set enrichment analysis (GSEA; https://www.gsea-msigdb.org) was performed on all expressed genes in the metastatic lesion (MET) and related organoid (PDO) samples. GSEA was run in preranked mode using classic as a metric and 1000 permutations selecting the curated gene sets of the Molecular Signatures Database (MsigDB) derived from the Hallmark and KEGG collections. As a ranking metric, the z scores of the genes were evaluated in each sample. Gene set enrichment was assessed through the normalized enrichment score (NES). The size of the circles reflects the percentage of genes in the core enrichment of the pathway. **H** Representative bright-field microscopy images of organoids generated from brain metastatic lesions carrying PIK3CA mutations treated with 10 µM alpelisib for 7 days. Magnification: 10X. The number of organoids and relative area for each condition are plotted in the bar graphs. **I** Flow cytometry of Helix NP blue-stained organoids generated from brain metastatic lesions carrying *PIK3CA* mutations after 7 days of treatment with 10 µM alpelisib. **J** Representative bright-field microscopy images of organoids generated from brain metastatic lesions with wild-type *PIK3CA* treated with 10 µM alpelisib for 7 days. Magnification: 10X. The number of organoids and relative area for each condition are plotted in the bar graphs. **K** Flow cytometry of Helix NP blue-stained organoids generated from brain metastatic lesions with wild-type *PIK3CA* after 7 days of treatment with 10 µM alpelisib

An 82-year-old woman was diagnosed in 2018 with invasive ductal carcinoma (IDC) of the right breast, grade 3, ER-/PR-/HER2 3 + , that had metastasized to the lymph nodes and bone. In 2019, contrast-enhanced brain computed tomography (CT) revealed a 3.9 cm cerebellar lesion that was surgically removed (Fig. 1A-C). We derived organoid cultures from this brain metastatic material (mOGs) by using a slightly modified organoid growth medium (OGM) containing neuregulin-1 to match the aberrant expression of ERBB family members in the starting material (Fig. 1D) [5, 6]. Using NGS-customized panels containing breast cancer-specific hotspot regions for 63 genes, we identified the *PIK3CA* H1047L mutation in both the patient-derived organoid (PDO) and the metastatic sample tissue, with variant allele frequencies (VAFs) of 45.4% and 56%, respectively (Fig. 1E and Suppl. Table 1). *PIK3CA* hotspot mutations drive oncogenic progression in several cancer settings [4]. These mutations occur in 30–40% of breast cancers and are associated with resistance to therapy and metastatic progression [7, 8]. We found that a very minor fraction of genes assessed in the mBC-PDOs exhibited different expression levels when compared with the brain metastasis sample of origin (Fig. 1F). This finding strongly validated the accuracy of the mBC-PDO model. Additionally, mutational analysis and Gene Set Enrichment Analysis (GSEA) of the paired metastatic lesion and the mBC-PDOs revealed that PI3K-related pathways were enriched (Fig. 1G).

We first evaluated 105 genes representative of the PI3K pathway by RNAseq (MSigDB, https://www.gsea-msigdb.org) to assess the relationship between the mutation status of *PIK3CA* and the activation of the PI3K pathway. We found the levels of PI3K genes to be significantly higher in *PIK3CA*-mutant brain metastases than in *PIK3CA*-wild-type brain metastases; the latter sample was obtained from a 53-year-old woman diagnosed in 2019 with IDC of the right breast ER + /PR + /HER2- (Suppl. Fig. 1A and Suppl. Table 2). The difference in the levels of the PI3K-related genes was even more evident in the mPDO cultures (Suppl. Fig. 1B). We stained brain metastasis-derived PDOs with an anti-AKT-phosphoS473 antibody. The flow cytometry data analysis revealed an enrichment of AKT-phosphoS473-positive cells in the *PIK3CA* mutant PDOs compared with those in the *PIK3CA* wild-type PDOs. Altogether, these results suggest that the PI3K pathway was activated due to the experimental conditions (Suppl. Fig. 1C). Finally, a breast cancer TCGA cohort analysis further validated our results. In breast cancer samples bearing mutated *PIK3CA*, despite a nonsignificant modulation of the transcriptional levels of PI3K pathway genes, both AKT, AKT-phosphoS473, AKT-phosphoT308 and PRAS40-phosphoT246 protein levels were significantly increased compared to those in wild-type *PIK3CA* samples (Suppl. Fig. 1D-E).

To test the functional relevance of PI3K pathway activation in PIK3CA-mutant PDOs, we treated the organoids with alpelisib, a clinical trial grade PI3K-α inhibitor [9–11], at pharmacologically relevant doses. This compound affected the mBC-PDO number (images and graph) and size (area graph) and reduced the number of live cells in the PIK3CA-mutant mBC-PDOs, as assessed by flow cytometry (Fig. 1H-I). To further relate the efficacy of alpelisib treatment to the presence of the *PIK3CA* mutation, we similarly treated non-*PIK3CA*-mutated brain metastasis-derived PDO cultures (ctrl-mBC-PDO) with alpelisib. Treatment of the ctr-mBC-PDO cultures with 10 µM alpelisib had little to no effect on PDO number (images and graph) or size (area graph), and no changes in the number of live cells was observed by flow cytometry (Fig. 1J-K). Taken together, these findings further suggest the dependency of mBC-PDOs on aberrant PI3K-propagated signalling for their maintenance.

**Fig. 2:**
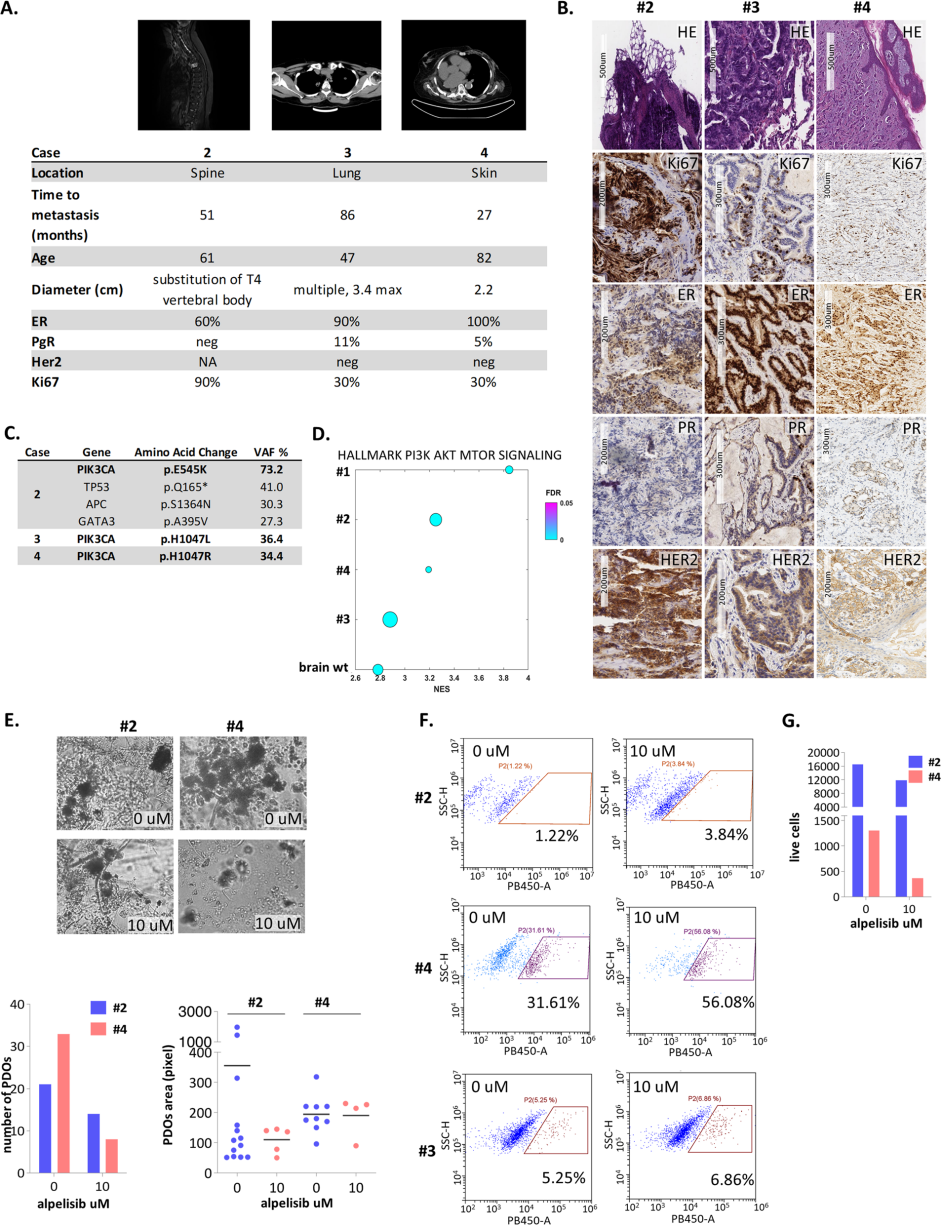
**A** Clinical features of cases 2, 3 and 4 metastatic lesions and relative magnetic resonance imaging (case 2) or computed tomography scans (cases 3 and 4). **B** Representative staining results from haematoxylin–eosin (HE) and immunohistochemistry analyses of ER, PR, HER2 and Ki-67 expression in the metastatic lesions from patients 2, 3 and 4. Scale bar = 200–500 µM. ER, oestrogen receptor; PR, progesterone receptor; HER2, human epidermal growth factor. **C** Summary of the mutational profile of metastatic lesions of cases 2, 3 and 4. VAF refers to variant allele frequency. **D** Normalized enrichment score (NES) of the PI3K pathway among the four patient cases harbouring *PIK3CA* mutations (#1, #2, #3, #4) and the brain metastasis with an intact *PIK3CA* gene. All expressed genes were ranked after z score transformation, and enrichment analysis was conducted for each sample. The size of the circles reflects the percentage of genes in the core enrichment of pathway. **E** Representative bright-field microscopy images of organoids generated from metastatic lesions of patients 2 and 4 treated with 10 µM alpelisib for 7 days. Magnification: 10X. The number of organoids for each condition is plotted in the bar graph. **F** Flow cytometry of Helix NP blue-stained organoids generated from metastatic lesions of patients 2, 3 and 4 after 7 days of treatment with 10 µM alpelisib. **G** Number of live cells of organoids derived from metastatic lesions of patients 2 and 4 after 7 days of treatment with 10 µM alpelisib

These encouraging findings prompted us to expand those observations to three additional cases of spine (case 2), lung (case 3) and skin (case 4) BC metastases, all with *PIK3CA* hotspot mutations. In detail, the case 2 sample was taken from a 61-year-old woman diagnosed in 2015 with pT2 pN3a IDC of the right breast, grade 3, ER + /PR + /HER2-, with a metastatic lesion to the spine (T4); the case 3 sample was taken from a 47-year-old woman diagnosed in 2014 with hormone-sensitive IDC breast cancer and multiple bilateral lung nodules, and the case 4 sample was taken from an 82-year-old woman with pT2 pN3 IDC of the left breast, grade 2, ER + /PR + /HER2-, with skin metastases on the left chest wall. All three patients had undergone previous Taxol and/or anastrozole treatment. The clinical and histopathological findings and representative magnetic resonance imaging (case 2) or computed tomography scans (cases 3 and 4) are shown in Fig. 2A. The receptor status, evaluated by immunohistochemistry, was ER + /PR-/HER2 2 + for the spine metastasis, ER + /PR + /HER2- for the lung metastasis and ER + /PR-/HER2- for the skin-derived metastasis (Fig. 2B), indicating changes in HR expression in the metastases versus that in the primary tumours. NGS tumour sequencing of the three metastatic lesions revealed the following *PIK3CA* hotspot mutations: E545K in case 2, H1047L in case 3 and H1047R in case 4 (Fig. 2C).

We next analysed the bulk RNA-seq data of the metastatic lesions by comparing the normalized enrichment score (NES) of the PI3K pathway between the four cases harbouring *PIK3CA* mutations and the brain metastasis with no *PIK3CA* gene mutations to validate our previous findings. All the expressed genes were ranked after z score transformation, and enrichment analysis was conducted for each sample. As reported in Fig. 2D, the metastatic lesions harbouring *PIK3CA* mutations exhibited effective enrichment for the PIK3-driven pathway when compared to the *PIK3CA* wild-type lesions. We supplemented the basic OGM (neuregulin 7.5 nM, EGF 10 ng/mL, FGF2 50 ng/mL, B27, BSA 2%) with factors specific for the host tissue to generate mBC-PDO cultures from the *PIK3CA*-mutated metastases as follows: KGF (20 ng/mL) for skin metastases; FGF2 (50 ng/mL) for spine-derived metastases and an inverted ratio of FGF2 and EGF for lung-derived metastases (FGF2 20 ng/mL, EGF 40 ng/mL). The numbers of formed PDOs from the spine, skin and lung metastases were counted, revealing the effective formation of aggregates within 72 h after media addition. Additionally, the area of the formed structures was measured over time and showed that the structures were actively expanding (data not shown). Finally, flow cytometric analysis of the disaggregated PDOs confirmed that most of the cells composing the PDO cultures were viable (data not shown). We followed untreated PDOs up to passage 5, witnessing a small significant decrease in their growth over time (data not shown). Notably, no PDO structures grew when the metastatic lesions were cultured with the medium used to generate the brain metastasis PDOs.

The three newly obtained mBC-PDO cultures were then challenged with alpelisib, and a variable yet significant decrease in PDO number (graph) and size (area graph) was observed; additionally, the number of live cells was reduced in the spine- and skin-derived mBC-PDOs (Fig. 2E-G). Lung metastasis-derived PDOs showed the most limited response to alpelisib. This reduced effect could be partly due to the lower enrichment for PI3K pathway-related genes, despite the presence of *PIK3CA* mutation, as identified by NGS tumour sequencing (Fig. 2D). This finding echoes the current knowledge regarding the heterogeneity of metastatic lesions (Fig. 2F). Finally, we expanded this limited cohort of samples to include an additional sample derived from skin metastasis in an 86-year-old patient diagnosed with invasive ductal carcinoma (IDC) of the right breast, grade 3, ER + /PR-/HER2, harbouring the wild-type *PIK3CA* gene (Suppl. Fig. 2A). A viability assay performed on the PDO cultures obtained from this sample treated with the control (DMSO) or alpelisib revealed little to no PDO-derived cell death in response to the drug. This finding again strengthens the link between the response to alpelisib and the presence of a *PIK3CA* gene mutation (Suppl. Fig. 2B).

## Discussion

Herein, we presented a test case evaluating six mBCs to demonstrate the possibility that an OGM based on tissue location may allow the effective growth and passage of mBC-PDOs. This test case is relevant because it may help address an unmet need in the field. The growth of organoids from breast cancer metastases has received less attention than the growth of organoids from primary tumours in recent years, as evidenced by the paucity of studies to date. The lack of studies may be due to the limited availability of metastatic material, as metastases are generally less amenable to surgical removal than primary lesions [12]; additionally, appropriate experimental conditions for PDO propagation are lacking. Our cohort of metastatic samples was also limited for these reasons. Growing metastatic material in OGM formulated for the primary tumour may not be ideal. Herein, we tested the hypothesis that if the metastatic material mimics the destination material, the formulation of OGM should be conducted based on the destination tissue instead of the “originating” tissue [13]. Additionally, we provided a test case in which mBC-PDOs from four patients retained sensitivity to alpelisib due to the *PIK3CA* mutation status of the originating lesion. Thus, we provide encouraging evidence that treating metastatic lesions based on the molecular status of the originating lesion may be logical and effective. Importantly, our reported findings require appropriate clinical studies for validation. TME components (cancer-associated fibroblasts, lymphocytes, macrophages) are rapidly underrepresented in epithelial organoids within a few passages [14, 15]. Therefore, we cannot evaluate the full contribution of the TME to the drug response. Future validation studies would greatly benefit from a multi-OMICs approach, including metabolomic, proteomic, transcriptomic and secretomic analyses. We believe that the data provided here establish a correlation between the genomic status of *PIK3CA* and PI3K pathway activation and provide initial evidence justifying such an approach. Nonetheless, the observations reported here still prove that metastatic material can be propagated in a way that mimics the bioarchitecture of the originating tissue by employing destination tissue-inspired media, thus allowing the evaluation of treatments targeted toward the molecular status of the lesion.

## Conclusion

In summary, our data reveal an unprecedented method of cultivating mBC-derived PDOs, with the subsequent possibility of interrogating such structures with limited pharmacogenomic screening in real time. Additionally, our findings indicate that lesion-based treatment, such as for *PIK3CA* gene mutations, may override multisite breast cancer metastatic specificity.

## Supplementary Information

**Additional file 1: Supplementary Figure 1.** *PIK3CA* mutation drives activation of the PI3K pathway. A. PI3K pathway-related genes were significantly upregulated in *PIK3CA*-mutated brain metastases compared to *PIK3CA* wild-type brain metastases. Distribution of the gene set expression included in the HALLMARK PI3K AKT MTOR SIGNALING pathway. Log2-transformed TPM values of the gene set were evaluated in the brain metastasis harbouring a *PIK3CA* mutation and in the wild-type brain metastasis. B. PI3K pathway-related genes were significantly upregulated in *PIK3CA-*mutated brain metastasis PDO cultures compared to in *PIK3CA* wild-type brain metastases. Distribution of the gene set expression included in the HALLMARK PI3K AKT MTOR SIGNALING pathway. Log2-transformed TPM values of the gene set were evaluated in the PDO of brain metastasis harbouring a* PIK3CA* mutation and in the wild-type brain metastasis. C. Activation of the PI3K pathway in brain metastasis-derived mPDOs. Briefly, brain mPDO-derived cells were starved of growth factors for 16 h before being fixed and permeabilized. Representative dot plots of single cells derived from brain metastatic PDOs bearing *PIK3CA* wild-type (left panels) or *PIK3CA* mutation (right panels) and stained with anti-AKT-pS473 (Cell Signalling, CA USA). Gates were drawn on unstained samples after excluding cell debris and doublets. D. Average expression level of genes involved in the HALLMARK PI3K-AKT-MTOR SIGNALING pathway. Normalized gene expression levels were obtained from the TCGA Breast Cancer dataset. Clinical data were downloaded from cBioPortal (cbioportal.org). We identified 317 *PIK3CA*-mutated patients and 658 *PIK3CA* wild-type patients. Overall, we did not observe any difference in the pathway between the two groups of patients at the gene level. E. *PIK3CA* mutation correlates with increased AKT, AKT-pS473, AKT-pT308 and PRAS40-pT246 protein levels in the TCGA Breast Cancer dataset. The box plots represent the distribution of protein expression from the TCGA Breast Cancer dataset. We obtained the expression levels of 223 proteins for 884 breast cancer patients from TCGA. The mutational status of *PIK3CA* was downloaded from cBioPortal (cBioPortal.org). Four proteins involved in the PI3K-AKT-MTOR SIGNALING pathway were found to be significantly upregulated in 253 *PIK3CA*-mutated patients compared to 539 *PIK3CA* wild-type patients.

**Additional file 2: Supplementary Figure 2.** A. Summary of the mutational profile of metastatic skin lesions. B. Flow cytometry of Helix NP blue-stained organoids generated from skin metastatic lesions harbouring wild-type PIK3CA after 7 days of treatment with 10 µM alpelisib.

**Additional file 3: Supplementary Table 1.** List of 63 genes included in NGS-customized panels.

**Additional file 4: Supplementary Table 2.** List of the genes (*n* = 105) representative of the PI3K pathway (MSigDB) considered in this study.

## Data Availability

All data generated or analysed during this study, if not included in this article and its supplementary information files, are available from the corresponding author on reasonable request.

## Abbreviations

BC: Breast cancer
mBC: Metastatic breast cancer
PIK3CA: Phosphatidylinositol-4,5-bisphosphate 3-kinase catalytic subunit alpha
PIK3-α: Phosphoinositide 3-kinase alpha
IDC: Invasive ductal carcinoma
ER: Oestrogen receptor
PR: Progesterone receptor
HER2: Human epidermal growth factor receptor 2
mOG: Organoid from metastases
OGM: Organoid growth medium
NGS: Next-generation sequencing
VAF: Variant allele frequency
PDO: Patient-derived organoid
mBC-PDO: Metastatic breast cancer patient-derived organoid
GSEA: Gene set enrichment analysis
TP53: Tumour protein p53
CT: Computed tomography
NES: Normalized enrichment score
RNA-seq: RNA sequencing
EGF: Epidermal growth factor
FGF2: Fibroblast growth factor 2
BSA: Bovine serum albumin
KGF: Keratinocyte growth factor
