# Supplementary figures and images for: A PIK3CA-mutant breast cancer metastatic patient-derived organoid approach to evaluate alpelisib treatment for multiple secondary lesions
Source: Mol Cancer. 2022 Jul 22;21:152. doi: 10.1186/s12943-022-01617-6 (PMC9306102; doi:10.1186/s12943-022-01617-6)

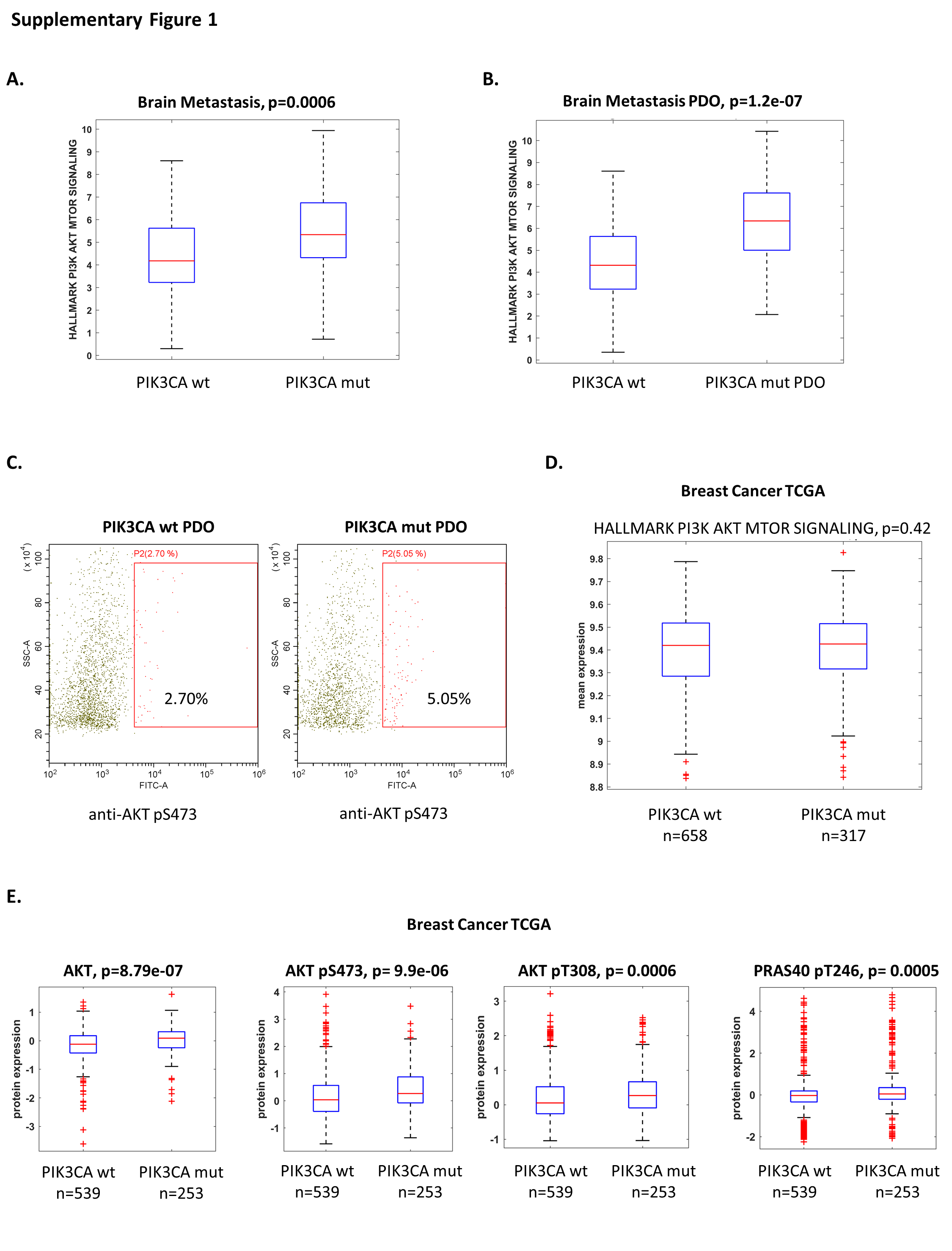

Supplement: Supplementary file 1 — Additional file 1: Supplementary Figure 1. PIK3CA mutation drives activation of the PI3K pathway. A. PI3K pathway-related genes were significantly upregulated in PIK3CA-mutated brain metastases compared to PIK3CA wild-type brain metastases. Distribution of the gene set expression included in the HALLMARK PI3K AKT MTOR SIGNALING pathway. Log2-transformed TPM values of the gene set were evaluated in the brain metastasis harbouring a PIK3CA mutation and in the wild-type brain metastasis. B. PI3K pathway-related genes were significantly upregulated in PIK3CA-mutated brain metastasis PDO cultures compared to in PIK3CA wild-type brain metastases. Distribution of the gene set expression included in the HALLMARK PI3K AKT MTOR SIGNALING pathway. Log2-transformed TPM values of the gene set were evaluated in the PDO of brain metastasis harbouring a PIK3CA mutation and in the wild-type brain metastasis. C. Activation of the PI3K pathway in brain metastasis-derived mPDOs. Briefly, brain mPDO-derived cells were starved of growth factors for 16 h before being fixed and permeabilized. Representative dot plots of single cells derived from brain metastatic PDOs bearing PIK3CA wild-type (left panels) or PIK3CA mutation (right panels) and stained with anti-AKT-pS473 (Cell Signalling, CA USA). Gates were drawn on unstained samples after excluding cell debris and doublets. D. Average expression level of genes involved in the HALLMARK PI3K-AKT-MTOR SIGNALING pathway. Normalized gene expression levels were obtained from the TCGA Breast Cancer dataset. Clinical data were downloaded from cBioPortal (cbioportal.org). We identified 317 PIK3CA-mutated patients and 658 PIK3CA wild-type patients. Overall, we did not observe any difference in the pathway between the two groups of patients at the gene level. E. PIK3CA mutation correlates with increased AKT, AKT-pS473, AKT-pT308 and PRAS40-pT246 protein levels in the TCGA Breast Cancer dataset. The box plots represent the distribution of protein exp [file 12943_2022_1617_MOESM1_ESM.tif]

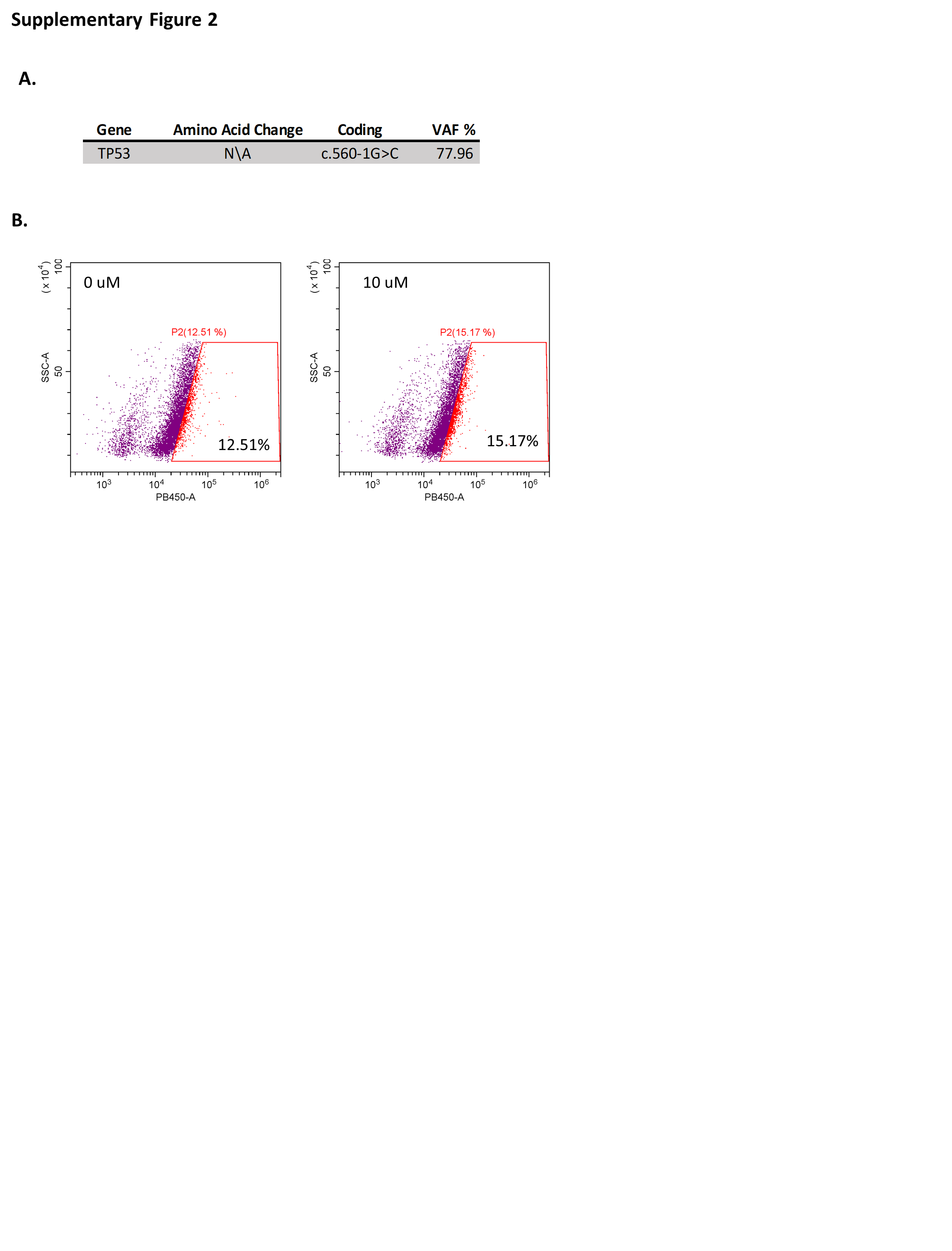

Supplement: Supplementary file 2 — Additional file 2: Supplementary Figure 2. A. Summary of the mutational profile of metastatic skin lesions. B. Flow cytometry of Helix NP blue-stained organoids generated from skin metastatic lesions harbouring wild-type PIK3CA after 7 days of treatment with 10 µM alpelisib. [file 12943_2022_1617_MOESM2_ESM.tif]
