# Supplementary material for: A PIK3CA-mutant breast cancer metastatic patient-derived organoid approach to evaluate alpelisib treatment for multiple secondary lesions
Source: Mol Cancer. 2022 Jul 22;21:152. doi: 10.1186/s12943-022-01617-6 (PMC9306102; doi:10.1186/s12943-022-01617-6)
Supplement: Supplementary file 3 — Additional file 3: Supplementary Table 1. List of 63 genes included in NGS-customized panels. [file 12943_2022_1617_MOESM3_ESM.docx]

**Supplementary Table 1.** *List of 63 genes included in NGS-customized panels.*
